# Injectable Click Fibroin Bioadhesive Derived from Spider Silk for Accelerating Wound Closure and Healing Bone Fracture

**DOI:** 10.3390/ma15155269

**Published:** 2022-07-30

**Authors:** Woong-Jin Lee, Kyoungjoo Cho, Aaron-Youngjae Kim, Gyung-Whan Kim

**Affiliations:** 1 Department of Neurology, College of Medicine, Yonsei University, Seoul 03722, Korea; osang0616@yuhs.ac (W.-J.L.); yok2019@qatar-med.cornell.edu (A.-Y.K.); 2 Department of Life Science, Kyonggi University, Suwon 16227, Korea; kcho0611@kgu.ac.kr; 3 Weill Cornell Medicine-Qatar, Doha P.O. Box 24144, Qatar

**Keywords:** bioadhesive, silk fibroin, wound closure, bone fracture

## Abstract

Wound closure is a critical step in postoperative wound recovery. Substantial advancements have been made in many different means of facilitating wound closure, including the use of tissue adhesives. Compared to conventional methods, such as suturing, tissue bioadhesives better accelerate wound closure. However, several existing tissue adhesives suffer from cytotoxicity, inadequate tissue adhesive strength, and high costs. In this study, a series of bioadhesives was produced using non-swellable spider silk-derived silk fibroin protein and an outer layer of swellable polyethylene glycol and tannic acid. The gelation time of the spider silk-derived silk fibroin protein bioadhesive is less than three minutes and thus can be used during rapid surgical wound closure. By adding polyethylene glycol (PEG) 2000 and tannic acid as co-crosslinking agents to the N-Hydroxysuccinimide (NHS), and 1-Ethyl-3-(3-dimethylaminopropyl) carbodiimide (EDC) reaction, the adhesive strength of the bioadhesive became 2.5 times greater than that of conventional fibrin glue adhesives. Silk fibroin bioadhesives do not show significant cytotoxicity in vitro compared with other bioadhesives. In conclusion, silk fibroin bioadhesive is promising as a new medical tool for more effective and efficient surgical wound closure, particularly in bone fractures.

## 1. Introduction

The use of medical tissue adhesives is an effective method for wound closure. Tissue adhesives are better for wound sites because they cause fewer secondary wounds than conventional sutures or staplers and cause less pain. There are several important characteristics of an ideal bioadhesive: (1) biocompatibility and degradability in vivo, (2) minimal toxicity or hypersensitivity, (3) flexibility in adhesion time for a variety of uses, (4) adequate adhesive strength of the solidified bioadhesive, (5) appropriate preparation time for its use during surgery, (6) low swelling index, (7) stability during storage, (8) adjustable decomposition time, (9) conspicuousness during surgery, and (10) simple application without special equipment or additional chemicals [1,2,3].

In this study, we introduced a bioadhesive containing silk fibroin protein derived from spider silk with PEG2000 (polyethylene glycol with a molecular weight of 2000) and tannic acid as a co-crosslinking agent. Surgical rejoining of damaged tissue is essential for restoring normal tissue structure and function [4,5]. Mechanical bonding materials, such as sutures, staplers, and external ears, have been conventionally used during surgical rejoining. The main purpose of bonding material is to join the tissues as closely as possible to prevent leakage of body fluids while simultaneously withstanding the tensile load exerted on the tissues [6,7,8]. However, the conventional methods have several disadvantages [9]. Sutures can cause trauma to the application site and surrounding tissues and might also prevent the normal flow of blood, body fluids, and air. In addition, sutures have poor accuracy for target tissues that are not easily accessible [10,11].

Silk fibroin from spider silk has a unique characteristic; the ingredient itself is effective in wound closure and bone regeneration [12]. The advantages of silk fibroin are: it increases the hemostatic effect by adhering to the surrounding bleeding site, mass productivity, biodegradability, wound regeneration function, bio-adhesion function, excellent biocompatibility, and is biohazard free. Tannic acid, used as an additive to the proposed adhesive, is one of the widely available polyphenols in the plant kingdom [13]. Early research on tannic acid had focused on physiological functions, such as antioxidants [14]. However, recently, it has been revealed that tannic acid binds to almost all biopolymers, such as proteins and DNA, and is receiving much attention as a molecular adhesive [15]. Various properties of tannic acid not only control the function and wettability of the surface, but also contribute to energy storage and generation devices, and show various possibilities as a medical agent [16]. In this study, the binding of tannic acid to biopolymers as a bioadhesive agent, surface modification through tannic acid, and its use as a medical agent are investigated. Based on the fact that the new material hydroxyapatite (HAP) has the same compositional structure as human bones and teeth, an attempt was made to synthesize a bone-specific bioadhesive [17]. HAP has the property of adsorbing to proteins, thus it is effective as a component of bioadhesives.

Many studies and developments have been made on tissue adhesives as a promising alternative to bonding materials. A recent report introduced the application of biomedical magnesium alloy by coating it [18]. In general, tissue adhesive refers to a material that can be polymerized to bond tissue and non-tissue (prosthetic implant) surfaces [19,20,21]. Tissue adhesives have several notable features. They have excellent hemostatic and air leakage prevention effects, lower risk of exposure to needles, easy removal after surgery, shortened operation time, and reduced side effects such as infection. Recently, the development of new tissue adhesives that can be used for various purposes has gained traction in the industry and research [22]. In this study, we used click chemistry reaction, which is commonly used in bioconjugation allowing the joining of substrates of choice with specific biomolecules. We demonstrated the synthesis and effectiveness of a web-derived silk fibroin bioadhesive based on click chemistry for rapid wound closure and bone fracture healing.

## 2. Materials and Methods

### 2.1. Chemicals and Reagents

Tannic acid, citric acid, chondroitin-6-sulfate, N-Hydroxysuccinimide (NHS), and 1-Ethyl-3-(3-dimethylaminopropyl) carbodiimide (EDC) were purchased from Sigma-Aldrich (St. Louis, MO, USA). Cobweb-derived silk fibroin was collected directly from spider silk living in South Korea. Sericin (Sigma, New York, NY, USA) was dissolved in 9.3 M LiBr (Sigma, New York, NY, USA) and dialyzed against distilled water using a 3500 MWCO dialysis membrane. They were then freeze-dried at −86 °C and used in this study.

A citric acid derivative (CAD) that is a precursor of silk fibroin protein bioadhesive was synthesized via an NHS/EDC reaction: carbodiimide crosslinking with the primary hydroxyl group of citric acid. Briefly, 0.04 g chondroitin-6-sulfate was dissolved in 35 mL of N,N-dimethyl formamide (DMF; Sigma, New York, NY, USA). Then, 1.9 g of NHS was dissolved in the chondroitin-6-sulfate solution. EDC (2.5 g) was dissolved in DMF (5 mL) and stirred with the previously prepared solution at room temperature for 24 h. One gram of citric acid in the above solution was stirred for 12 h and lyophilized to obtain CAD.

**Figure 1:**
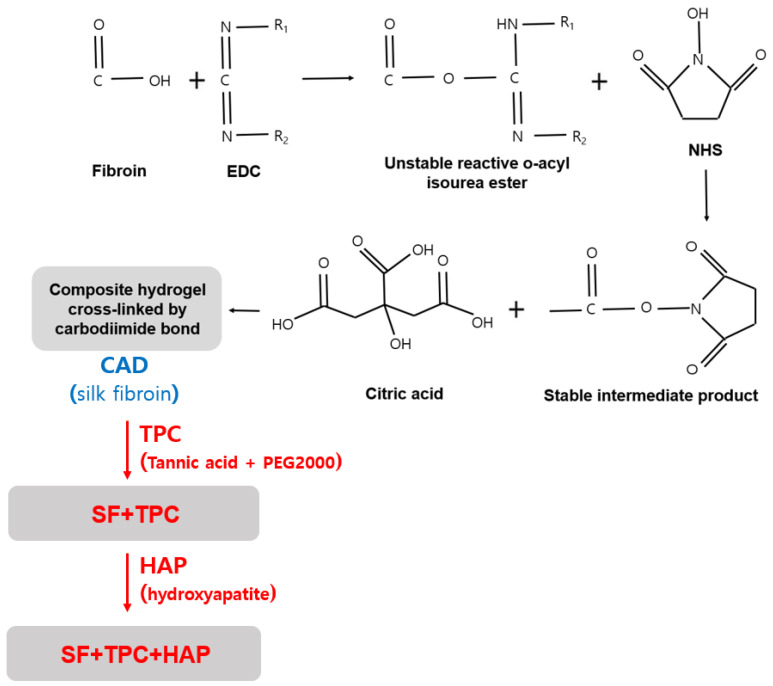
A schematic summary of two bioadhesives.

Tannic acid and polyethylene glycol 2000 (PEG 2000, Sigma, New York, NY, USA) curing agents (TPC) were synthesized through the following process: tannic acid (Sigma, New York, NY, USA) was dissolved in 1 × PBS (pH 8.5) at 1 g/mL, and PEG 2000 was dissolved in 1 × PBS at 1 g/mL. Pyrogallol: PEG (functional group) was obtained by mixing tannin and PEG in a 25:1 molar ratio. After 10 min of reaction, the supernatant was removed and freeze-dried at −8 °C and the precipitate was used to obtain TPC. When using the silk fibroin bioadhesives, 400 µL, 130 mg CAD solution, and 44% of spider silk fibroin protein was added to PBS. Then, 50% (*w*/*v*) TPC was mixed with the previously prepared solution. In this study, we introduced two kinds of adhesives. One is CAD, a precursor of silk fibroin (SF), by adding TPC (SF + TPC), and the other is ‘SF + TPC’ with addition of HAP (SF + TPC + HAP). These adhesives are displayed in Figure 1.

### 2.2. GC-MS Component Analysis

Gas chromatography/mass spectroscopy (GC-2010; Shimadzu Co., Kyoto, Japan) was used to observe the spider silk-derived silk fibroin protein components. After each extract was dissolved in ethanol, the suspension was removed using centrifugation and filtered through a fine filter (0.45 μm). Component analysis was performed under the following conditions:

(1) Column was BD-5 (60 mm × 0.25 mm × 0.25 mm),
(2) Carrier gas was He (1 mL/min),
(3) Injection temperature was 250 °C,
(4) Oven temperature was set to 50–300 °C with a 3 K/min rise,
(5) Injection volume was 1 μL.

The injection mode was analyzed at a split ratio of 10:1. The mass range was 28–550 u in a mass selective detector and the acquisition mode was quantified under scan mode conditions. Finally, compound identification was performed by matching with the Wiley library [23].

### 2.3. Determination of Constituent Amino Acid Content

We weighed 0.5 g of the sample powder into an 18 mL test tube, added 3 mL of 6 N HCl, and sealed the test tube using a vacuum pump. After hydrolyzing the sealed test tube at 121 °C for 24 h on a heating block, the acid was removed using a rotary evaporator at 50 °C and 40 psi. The test tube content was then filtered through a membrane filter (0.2 μm) with 1 mL of sodium loading buffer solution. The filtered solution was quantitatively analyzed using an amino acid analyzer (S433-H, SYKAM, Eresing, Germany) with the following specifications: the column used was a cation separation column (LCA K06/Na) with a column size of 4.6 × 150 mm and a column temperature of 57–74 °C; the concentrations of buffer and reagent were 0.45 mL/min and 0.25 mL/min, respectively, and the flow rate was 0.25 mL/min. The wavelengths were 440 and 570 nm for the buffer and the reagent, respectively.

### 2.4. Determination of Chondroitin-6-Sulfate Content

Chondroitinase (Sigma, Taufkirchen, Germany), Waters’ 515 pump, controller, and 486 UV detector were used for HPLC analysis to measure chondroitin-6-sulfate content. The fibroin bioadhesive was prepared and inserted into the columns for analysis using a Phenomenex Luna C18. Silk fibroin protein extracts were analyzed using HPLC, and a graph comparing the concentrations of chondroitin-6-sulfate in silk fibrin (negative control), silk fibroin protein, and silk fibroin protein with hydroxyapatite was plotted. MeOH:KH_2_PO_4_ = 1:4 *v*/*v* was used as the mobile phase, and 0.1, 0.2, 0.4, and 0.8 wt% of samples and the standards were dissolved in HPLC water for analysis. The area was calculated based on the concentration.

### 2.5. FTIR Structural Analysis

The extract was analyzed using infrared spectroscopy (FTIR spectroscopy, Shimadzu-IR Prestige-21, Shimadzu Co., Kyoto, Japan) to determine its chemical structure. First, the specimens were prepared by mixing the samples with KBr at a weight ratio of 1:100. After removing moisture by drying under reduced pressure at 60 °C for 12 h, the extract was measured in the 4000–500 cm^−1^ area.

### 2.6. Contact Angle Measurement

The contact angle is affected by the surface tension between the liquid and solid. It is primarily used to measure the wettability of a solid surface. The lower the contact angle, the greater the hydrophilicity, and vice versa. To identify the hydrophilic and hydrophobic properties of silk fibroin protein derived from the spider silk, 5% of each extract was dissolved in distilled water, poured into a Petri dish (150 mm), and dried at 50 °C for 4 h. The contact angle between the water droplet and cobweb-derived silk fibroin protein membrane was measured. Using a contact angle measuring device (Phoenix-Smart (A), Surface Electro Optics, Suwon, Korea), each measurement was performed five times and the average value was calculated.

### 2.7. Adhesive Strength Measurement

Each protein extract was concentrated ten times, and the adhesive strength was measured using universal testing machine (UTM) equipment [24]. Pig skin was purchased from a slaughterhouse and used to evaluate the adhesive strength of the silk fibroin protein extract derived from spider silk. After washing with sterile distilled water, the pig skin was wrapped in foil, kept at −20 °C, and used within 4 weeks. The pig skin was then cut into two 25-mm wide and 70-mm long pieces and placed such that they overlapped 25 mm along their lengths. Different types of bioadhesives were applied between the overlapping regions. Eventually, the ASTM D5868-01 specifications were used to pull and measure the strength that each bioadhesive can withstand before each side of the pig skin is separated from the other. In detail, we prepared 0.2 mL of the bioadhesive and applied it to the surface of the pig skin (1 × 1 cm^2^). After joining pig skins of the same size, a load of 50 g was applied and left for 10 min to harden the gel. The load was removed after 10 min, and a shear force was applied continuously using a tensile tester (H5K-T, Hounsfield) at a rate of 100 mm/min until the adhered pig skins were peeled off from each other.

### 2.8. Cytotoxicity

The stability of the spider silk-derived silk fibroin protein composition adhesive was evaluated according to the method presented in ISO 10993-5. L-929 fibroblasts purchased from the Korean Cell Line Bank (KCLB) were cultured in DMEM medium containing 10% FBS and 1% penicillin at 37 °C and 5% CO_2_. CCK assay was performed by treating a plate cultured at 5 × 10^4^ cells/well for 24 h with the protein composition adhesive eluted in the medium at a high concentration. After measuring the absorbance at 570 nm using an ELISA reader (Biotex, Pearland, TX, USA), the cell viability was calculated. Each experiment with fibrin glue, fibroin + TPC, and fibroin + TPC + hydroxyapatite (HAP) was performed thrice.

### 2.9. Assessment of Gelation Times

The gelation time was tested using a test-tube tilting method, as reported previously [25]. A total volume of 100 μL of fibrin glue, silk fibroin, and the solution was added to a glass tube with different formulations. The tube was shaken gently, and the time until the solution lost mobility was recorded. The experiments were conducted thrice at room temperature.

### 2.10. Alkaline Phosphatase Assay

To investigate the effect of the SF + TPC + HAP bioadhesive on osteogenic differentiation, the activity of alkaline phosphatase (ALP), an early marker of osteogenic differentiation, was analyzed [26]. After seeding 3 × 10^4^ cells/cm^2^ of saos-2 cells in a 24-well plate, 10 mg of each bioadhesive agent (fibrin glue, SF + TPC, SF + TPC + HAP) was administered to obtain a concentration with negligible cytotoxicity. These solutions were then administered and cultured to induce osteocyte differentiation for 5 days to analyze ALP activity. Briefly, cells were lysed using NP40 cell lysis buffer (Life Technologies, Carlsbad, CA, USA). In some of the cell lysates obtained from this process, the buffer and p-nitrophenyl phosphate (Thermo Fisher Scientific, Waltham, MA, USA), a substrate of ALP, were added, and the enzymatic reaction was performed for 20 min at 37 °C. After terminating the reaction by adding 0.9 N NaOH solution, the ALP activity was analyzed by measuring the absorbance at a wavelength of 405 nm using a microplate reader (Molecular Devices, San Jose, CA, USA). The final ALP activity was determined by calculating the absorbance for the same amount of protein in the cell lysates to adjust the absorbance according to the number of cells.

### 2.11. Statistics

All results are reported as mean ± standard error. Student’s *t*-tests were performed to compare the differences between groups, including an unpaired *t*-test for cell viability under different treatments and paired *t*-test. Statistical significance was set as *p* < 0.05.

## 3. Results and Discussion

### 3.1. Analyzing the Constituents and Amino Acid Contents in Fibroin Protein

Amino acid compositions of fibrin and silk fibroin used in this study are listed in Table 1. Amino acid content per 100 g of fibrin powder had high amounts of glutamic acid (32%), aspartic acid (25.9%), and valine (16.8%). In fibroin, glycine (44.1%), alanine (31.3%), and serine (12.5%) were predominant (Table 1). GC-MS peaks showed that the components of fibrin glue and silk fibroin samples contained more than 85% of glycyl-L-proline (C_7_H_12_N_2_O_3_) (Table 2). In addition, palmitic acid, propenoic acid (unsaturated liquid carboxylic acid), and cyclopentane were detected in the fibrin and silk fibroin groups. Silk fibroin has significantly higher contents of glycine, alanine, and serine compared to that of fibrin protein. However, silk fibroin protein has significantly lower contents of valine, aspartic acid, and glutamic acid.

### 3.2. FTIR Structural Analysis

**Figure 2:**
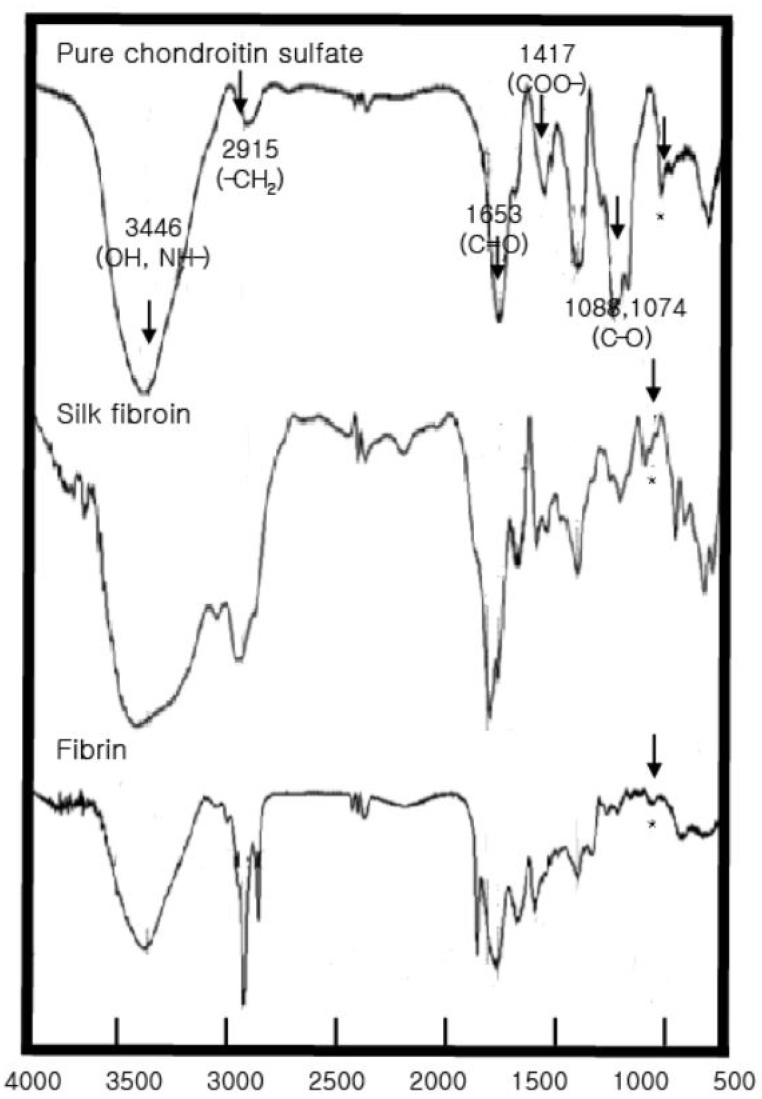
FTIR analysis of pure chondroitin 6-sulfate, silk fibroin, and fibrin. Peaks indicate specific chemical bonds. The peak near 850 cm^−1^ (*) represents the C-O-C structure of glucose, galactose, and mannose, indicating the characteristics of monosaccharides.

To analyze the bioadhesives’ structural characteristics, the FTIR spectrum of the chondroitin sulfate standard showed large peaks at 3446.79, 1643.35, 1251.8, 1064.71, and 994.43 cm^−1^ and medium-sized peaks at 1423.52, 1148.36, 809.50, and 586.36 cm^−1^ (Figure 2). The peak near 850 cm^−1^ (*) represents the C-O-C structure of glucose, galactose, and mannose, indicating the characteristics of monosaccharides. For the gelatin standard, large peaks were observed at 3425.58, 1649.14, 1246.02, 601.36, and 559.36 cm^−1^, and medium-sized peaks were observed at 2887, 1544, and 1449 cm^−1^.

For fibrin glue and silk fibroin, FTIR analysis showed large peaks at 3468.01, 2968.45, 1689.64, 1242, and 18,601.79 cm^−1^ and medium-sized peaks at 1650.32, 1120.11, 984.04, and 850,750.25 cm^−1^. These peaks correspond to gelatin. In particular, functional groups of amino acids and proteins, including amides I and II, were identified in the 1700–1500 cm^−1^ region. Furthermore, 1652 cm^−1^ represents amide I, 1688 and 1638 cm^−1^ represent antiparallel β-sheets, and 1649 cm^−1^ reflects a random structure. The peaks at 1663–1630 cm^−1^ are characteristic of the amide I band, 1738 cm^−1^ is a lipid, and 2900 cm^−1^ (CH_2_, amide B) and 3400–3440 cm^−1^ (amide A) are amide-N and amide A, respectively. In addition, the peak at 850 cm^−1^ (*) indicates the chondroitin sulfate component, which was also observed in the fibrin glue and silk fibroin FTIR analysis.

### 3.3. Adhesive Strength and Contact Angle Measurement

**Figure 3:**
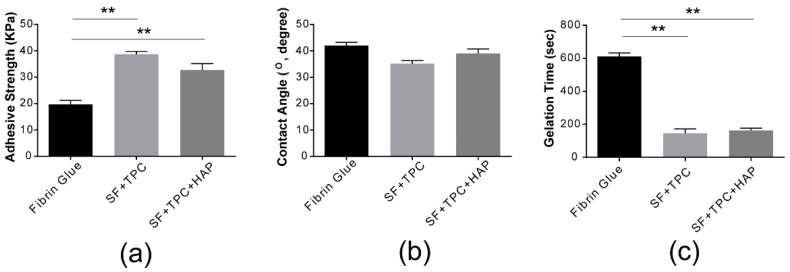
Structural analysis. (**a**) The adhesive strength of conventional fibrin glue was compared to silk fibroin protein in skin (SF + TPC) and in bone (SF + TPC + HAP). Silk fibroin showed about two times higher adhesive strength than conventional fibrin glue. (**b**) The contact angle of conventional fibrin glue was compared to silk fibroin protein in skin (SF + TPC) and in bone (SF + TPC + HAP). Overall, SF showed a lower contact angle compared to fibrin, suggesting greater hydrophilicity. (**c**) The gelation time. SF, silk fibroin; TPC, tannic acid and polyethylene glycol 2000 as curing agents; HAP, hydroxyapatite. *n* = 5. ** *p* < 0.001.

As shown in Figure 3a, our silk fibroin bioadhesive has greater strength than conventional fibrin glue. The adhesive strength of conventional fibrin glue was compared to that of silk fibroin protein in skin (SF + TPC) and bone (SF + TPC + HAP). Silk fibroin showed approximately two times higher adhesive strength than conventional fibrin glue. For effective contact, the adhesive must be wetted by the adherend. Wetting occurs when the bonding force at the interface between the adhesive and adherend molecules is greater than the cohesive force between the molecules of the adhesive [27,28]. Wettable compounds or surfaces have a contact angle <90°. The composition of the SF + TPC used in the present study had higher hydrophilic properties, with a film composition of 41 ± 3° and 35 ± 2° for the fibrin glue composition. This composition can infer the proposed adhesives with a more wettable characteristic, which provide higher cohesive force to attach skins than conventional fibrin glue. The contact angle of the SF + TPC + HAP composition film was 38 ± 7°, suggesting higher hydrophilicity than that of fibrin glue (Figure 3b). The gelation time of fibrin was approximately five minutes (Figure 3c). Compared to fibrin, silk fibroin showed a shorter gelation time of less than three minutes, making it a better candidate for new bioadhesives.

### 3.4. Cytotoxicity and ALP Activity

**Figure 4:**
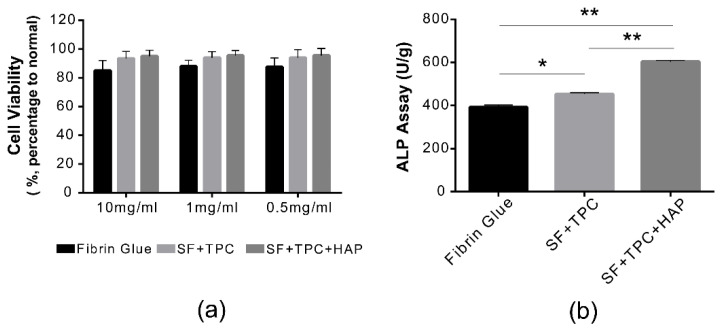
Functional analysis. (**a**) Cell viabilities were measured in both undiluted (10 mg/mL) and diluted states (1 mg/mL and 0.5 mg/mL) of both fibrin and silk fibroin proteins. There was no difference in cell viability among conventional fibrin glue, silk fibroin protein in skin (SF + TPC), or silk fibroin protein in bone (SF + TPC + HAP). There was no cytotoxicity at 10 mg/mL. (**b**) ALP activity was presented as U/g in each group of fibrin, SF + TPC, and SF + TPC + HAP. SF, silk fibroin; TPC, tannic acid, and polyethylene glycol 2000 as curing agents; HAP, hydroxyapatite. *n* = 5, * *p* < 0.05; ** *p* < 0.001.

The cytotoxicity of fibrin glue, SF + TPC, and SF + TPC + HAP adhesives was evaluated (Figure 4a). The most important characteristic of adhesive glue is definitely an adhesive ability. When the adhesives are applied to human skin or organs, the adhesives are safe. We tested the safety on live cells. Overall, a survival rate ≥80% or more was observed for each composition. In the case of the fibrin glue adhesive selected as a control, the cell viability was 78.2 ± 10% at 10 mg/mL, showing an increase of over 80% at other concentrations. For fibroin glue adhesive, cell viability was 86 ± 12.23% at 10 mg/mL, and higher viability in other diluted states. As both fibrin and fibroin glues showed higher cell viability than cyanide acrylate (70% cell viability), fibroin protein can be readily applicable to human tissues and bones [29,30]. To investigate the effect of SF + TPC + HAP on cellular activity, ALP activity in cells was measured. Compared with the control group that used conventional fibrin glue, ALP activity significantly increased in the SF + TPC and SF + TPC + HAP treatment groups (Figure 4b). These experimental results suggest that SF + TPC + HAP treatment promotes the initial process of cell survival and differentiation.

## 4. Conclusions

In this study, a bioadhesive was prepared using a silk fibroin protein derived from spider silk. Silk fibroin (SF) protein was studied for its potential use as a medical bioadhesive. We also quantified chondroitin sulfate using HPLC and FTIR analysis. A two-fold higher amino acid content was observed in the fibroin extract than that in the fibrin extract. The adhesive strength test using pig skin also showed two-fold higher adhesive strength of the silk fibroin compared to the fibrin protein. Measurement of cell cytotoxicity of the silk fibroin bioadhesive showed low cytotoxicity, suggesting that it is a promising alternative to fibrin glue for wound closure and bone fracture healing.

The new bioadhesives are injectable and have a fast gelation time and minimal cell cytotoxicity. Moreover, they have stronger adhesion strength to tissues and facilitate better wound healing than fibrin glue. The accelerated healing outcomes could possibly be due to the ability of the new bioadhesive to effectively close and reduce the tissue gap. On the other hand, collagen can be introduced into the system and form a dual network with silk fibroin bioadhesives, increasing the adhesive strength.

## Figures and Tables

**Table 1:** Amino acid composition of fibrin and fibroin extract.

Amino Acid Component (g/%)	Fibrin	Fibroin
Glycine	2.4	44.1
Alanine	7.4	31.3
Serine	10.4	12.5
Tyrosine	3.2	4.9
Valine	16.8	2.5
Aspartic acid	25.9	1.9
Glutamic acid	32	1.7
Threonine	1.9	1.1
**Total**	**100**	**100**

**Table 2:** Gelation time(s) of fibrin, SF + TPC, and SF + TPC + HAP.

Name of Bioadhesives	Gelation Time (s)
Fibrin glue	611 ± 22
SF + TPC	146 ± 26
SF + TPC + HAP	163 ± 14

SF, silk fibroin; TPC, tannic acid and polyethylene glycol 2000 as curing agents; HAP, hydroxyapatite.

## Data Availability

Not applicable.
